# Variations in Microcirculatory and Hemodynamic Parameters during Oncological Demolitive–Reconstructive Head and Neck Surgery: A Protocol for an Observational Study

**DOI:** 10.3390/mps6040067

**Published:** 2023-07-21

**Authors:** Chiara Adembri, Andrea Ungar, Iacopo Cappellini, Salvatore Mario Romano

**Affiliations:** 1 Department of Health Sciences, Section of Anesthesiology, University of Florence, 50121 Florence, Italy; chiara.adembri@unifi.it; 2 Geriatric Intensive Care Unit, University of Florence, 50121 Florence, Italy; andrea.ungar@unifi.it; 3 Department of Critical Care, Section of Anesthesiology and Critical Care Azienda USL Toscana Centro, Ospedale Santo Stefano, 59100 Prato, Italy; 4 Unit of Internal Medicine and Cardiology, Department of Experimental and Clinical Medicine, University of Florence, 50121 Florence, Italy; salvatore.romano@unifi.it

**Keywords:** free flap surgery, microcirculation, macrocirculation

## Abstract

(1) Background: Oncological demolitive–reconstructive surgeries in the head and neck region cause significant stress on patients’ biohumoural, cardiac, and vascular systems, leading to disturbances in macrocirculatory and microcirculatory parameters. Traditional monitoring addresses the symptoms, but not the underlying cause. Microcirculatory assessments complement macrocirculatory monitoring, and bladder-catheter-based technology offers a better representation of central microcirculation. Flap reconstruction surgeries involve demolitive and reconstructive phases, requiring optimal tissue perfusion. The literature lacks a consensus on macro–microcirculation coupling, and there is no agreement on the use of vasopressors during head and neck surgeries. Evidence-based guidelines are lacking, resulting in variations in vasopressor administration. (2) Methods: This is a 12-month observational, prospective study conducted in a single center. It aims to evaluate the impact of macro–microcirculation coupling on clinical complications in head and neck surgery. All consecutive patients undergoing oncologic surgery requiring flap reconstruction and meeting the inclusion criteria will be enrolled. The study will utilize standard hemodynamic monitoring and bladder catheterization for measuring urine output and temperature. (3) Conclusions: The study aims to evaluate the coupling of macro- and microcirculation in head and neck surgeries, assess hemodynamic parameters and microcirculatory changes, and investigate their association with postoperative complications. The results can enhance patient care and surgical outcomes.

## 1. Introduction

Major oncological demolitive–reconstructive surgeries of the head and neck region are characterized by prolonged operating times, during which patients experience significant biohumoural, cardiac, and vascular stress. This can lead to disturbances in both macrocirculatory and microcirculatory parameters. While these alterations are often addressed by administering fluids and vasoactive medications to correct the observed events, the underlying cause remains unaddressed. To better understand and potentially prevent these events, it is important to complete traditional macrocirculatory monitoring with microcirculatory assessment [1].

Various devices, such as pulse contour monitors (PCM), are currently available for macrocirculatory monitoring with minimal impact on patient stress. Generally, PCMs use the calibrations and/or the pre-calculate data to obtain the flow of the patients [2,3,4]. On the other hand, in order to obtain the flow we will utilize a specific device that relies solely on the morphology of the patient under study, without the need for calibrations or pre-estimated data from other patients. This device enables us to analyze the morphology of the arterial waveform, focusing solely on cannulating a peripheral artery without considering other parameters [5,6,7,8]. However, monitoring the peripheral microcirculation is more challenging and the interpretation of data from different techniques is difficult due to variations depending on the examined area (e.g., sublingual, fingers, ears). Recently introduced technology utilizes a bladder catheter with a sensor in contact with the urethra to evaluate the microcirculation. This approach may provide a better representation of the perfusion in the “central” microcirculation [9,10,11].

Demolitive surgery with flap reconstruction is the primary treatment for neoplastic conditions in the head and neck region. These procedures involve highly demolitive phases with intense nociceptive stimulation (e.g., bone resections), followed by reconstructive phases with microsurgery, where nociceptive stimuli are reduced, and tissue perfusion must be optimally maintained to ensure successful microvascular anastomosis.

The current literature lacks consensus regarding the coupling of macrocirculation with microcirculation, both for physiological and pharmacological confoundings [12]. Many microcirculatory assessment technologies are highly dependent on the specific site of measurement, and the effects of administered drugs can differ between central and peripheral levels [13,14,15]. In particular, this coupling is even less studied in the context of oncological demolitive–reconstructive surgery of the head and neck region, and there is currently no agreement on the use of intraoperative vasopressors. While vasopressors can improve systemic pressure, they may also reduce perfusion to flaps and other organs. Additionally, evidence-based guidelines derived from prospective studies are lacking. As a result, there is a wide range of practices regarding the administration and selection of vasopressors, based on the experience of individual centers and operators [16].

## 2. Experimental Design

This is an observational, prospective, single-center, non-profit study. All consecutive patients undergoing oncological head and neck surgery with free flap reconstruction within a 12-month period (starting from the date of ethical committee approval for the following 12 months) and meeting the inclusion criteria will be enrolled. The routine hemodynamic monitoring usually used for these type of surgery allows for the continuous recording of macrocirculatory parameters, including Pulse Rate Variability (PRV), Stroke Volume (SV), Cardiac Output (CO), Systemic Vascular Resistance (SVR), dp/dt max, and Cardiac Cycle Efficiency (CCE), using the Pressure Recording Analytical Method (PRAM) technique [17,18]. These parameters are calculated by analyzing beat-to-beat variations in arterial pressure waveforms obtained at a frequency of 1000 Hz from a standard cannula inserted into an artery (MostCare-UP^®^, Vygon, Ecouen, France). The monitoring of the measured variables will be performed using the radial access technique.

Additionally, all patients undergoing demolitive–reconstructive surgery require the placement of a bladder catheter, which will remain in place during the postoperative period. This catheter will allow for the measurement of urine output and the patient’s temperature. By using the IKORUS (IKORUS UP^®^, VYGON, Ecouen, France) catheter to monitor urine output and temperature, the continuous monitoring of the microcirculation will also be achieved.

All patients will spend at least the first postoperative day in the ICU, enabling monitoring to continue for at least 24 h in a controlled environment.

Patients will be asked to provide their consent to participate in the study, with a clear and straightforward explanation of the study’s objectives. It will be emphasized that participation will not involve any modification to their standard treatment and is entirely voluntary. The pharmacological treatment of participating patients will follow standard guidelines and will be identical to that of non-participating patients.

Setting: The study will take place in the operating rooms affiliated with the Anesthesia for Sensory Organs and Surgical Intensive Care Unit. Enrollment will commence after obtaining approval from the ethical committee. All patients undergoing oncologic surgery of the head and neck region requiring flap reconstruction will be included in the study if they provide informed consent and meet the inclusion criteria.

Inclusion criteria: Patients undergoing oncologic surgery of the head and neck region requiring free flap reconstruction (oral cancers, tongue, etc.) who are admitted to the geriatric intensive care unit in the postoperative period. Age 18–85 yo, BMI 15–35, eligible for this type of surgery multidisciplinary oncologic evaluation, informed consent, ASA status <IV.

Exclusion criteria: Patients in whom cannulation of the peripheral artery would not be possible. Patients in whom the IKORUS catheter cannot be inserted (due to anatomical or pathological reasons) and who will require special catheters inserted directly by urologists. Patients who will not give their consent.

Study outcomes: The study aims to evaluate the coupling between macro- and microcirculation and its potential impact on the intra- and postoperative clinical outcomes. The clinical outcomes will be classified according to the Clavien–Dindo classification during the hospital stay [19]. Additional follow up will be performed at 3 and 6 months for the evaluation of cardiovascular and renal events.

Sample size: All eligible cases within the study criteria will be included over a 12-month period. It is estimated that approximately 30 patients will undergo free flap reconstruction.

### 2.1. Materials

#### 2.1.1. Study of Macrocirculation

For the definition of the hemodynamic parameters mentioned above, please refer to the literature on the subject, as they are well-known parameters [17]. Here are specific details for the following parameters.

Heart Rate Variability (HRV) is the fluctuation in time intervals between adjacent heartbeats generated by the interaction between the heart and the brain through the autonomic nervous system (sympathetic and parasympathetic), which acts dynamically and non-linearly. HRV is the result of interdependent regulatory systems that facilitate our adaptation to environmental and psychological variations [20,21].

PRV is the oscillation in time intervals between adjacent blood pressure waves, as the RR interval variations observed in HRV correspond to variations in the duration of the individual cardiac cycle described by the arterial pressure wave (pressor cardiac cycle). Some studies have compared these two methods of studying cardiac rhythm variability and confirmed a substantial correspondence between the two methods [20].

CCE is a more recently introduced and less well-known hemodynamic parameter. It is an index of hemodynamic performance that reflects the energy expenditure sustained by the cardiovascular system (heart and circulation) to maintain a specific hemodynamic state. It is expressed as a number (ranging from +1 to −1) derived from the ratio between the work performed by the system and the energy expenditure necessary to maintain adequate homeostasis. Since no machine has 100% efficiency, no individual has a CCE equal to +1. To simplify, young and healthy individuals will have positive CCE values, older individuals will have lower positive values, and decompensated elderly individuals will have negative CCE values [17].

The MostCare-up system allows for the real-time display of variables related to both macrocirculation and tissue perfusion. These variables are electronically acquired and stored within the device; this device records arterial pressure signals at a sampling rate of 1000 Hz. Beat-to-beat analysis is performed, and the results are saved in electronic format in the system’s internal memory. Two CSV files are generated, one containing the average values of the variables every 30 s, and the other containing the beat-to-beat values of all the variables displayed on the monitor. PRV values are recorded every 5 min in the file with the 30 s averages (as per standard requirements for beat-to-beat analysis). Additionally, the beat-to-beat CSV file stored within the MostCare-up device allows for the offline analysis of PRV.

The PCM method utilized in this study does not rely on external calibrations or pre-estimated data. It is implemented within the technology of the MostCare-up system, which electronically stores parameters such as pulse rate, arterial pressures, and flow. This enables the investigation of the relationship between macrocirculation and microcirculation, as well as the assessment of variations in the sympathetic–parasympathetic system through PRV. Both the macrocirculatory and microcirculatory systems are influenced by changes in the balance of the sympathetic–parasympathetic system induced by drugs and the surgical procedure.

Healthy biological systems exhibit complex, nonlinear oscillations in the intervals between consecutive beats and blood pressure waves, reflecting their adaptability to environmental changes. Diseases can disrupt this complexity, resulting in reduced HRV and PRV, which are generally indicative of pathological conditions. However, certain pathological conditions, such as intramyocardial conduction abnormalities, can increase HRV and PRV. Optimal levels of HRV and PRV are associated with good health status, autoregulatory capacity, adaptability, and resilience [17].

#### 2.1.2. Study of Microcirculation

For microcirculation monitoring, the IKORUS system (IKORUS UP^®^, VYGON, Ecouen, France) will be employed. Alongside routine measurements of bladder temperature and urine collection, this special catheter enables the real-time visualization of saturation variations using a photodiode. These variations closely correlate with microcirculatory changes at the urethro-vesical junction [9,10].

Assessing microcirculation poses a challenge in extrapolating internal organ conditions from a peripheral district. However, the IKORUS system offers the advantage of measuring parameters at a “central” level, with bladder temperature being considered a reliable reflection of core temperature. This provides an opportunity to interpret and study the microcirculation and the interplay between macro- and microcirculation as an integrated systemic response.

The IKORUS system incorporates a urethral probe featuring a photopletysmography sensor (PPG) positioned within a Foley catheter to monitor the urethral Perfusion Index (uPI). The PPG sensor makes direct contact with the urethral mucosa, capturing the pulsatile waveform. Employing an AC/DC ratio approach, the dedicated IKORUS monitor calculates the uPI, which demonstrates a close correlation with the standard perfusion index derived from pulse oximetry. The uPI represents the integral of pulsatile blood absorbance over a specific time window, yielding valuable insights into variations in total hemoglobin concentration weighted by tissue oxygen saturation. The monitor continuously displays the results of the uPI analysis at regular intervals of 10 s, 1 min, and 5 min, enabling the real-time monitoring of tissue perfusion.

This approach provides a novel means for studying microcirculation within a central domain, facilitating a comprehensive understanding of the relationship between macro- and microcirculation as an integrated systemic response. The IKORUS system’s capability to measure the uPI offers valuable insights into tissue perfusion dynamics, enhancing our knowledge of microcirculatory changes in various clinical scenarios [10].

In conclusion, the availability of minimally invasive hemodynamic monitoring systems, including PRV, based on PCM such as the upgraded version of MostCare-up, which is already in routine use in our institution for surgical patients, allows for the association of macrocirculatory variations, PRV, and hemodynamic profiles with the study of microcirculation using the IKORUS system during major demolitive–reconstructive surgeries in the head and neck region [6].

##### Statistical Plan

**Figure 1:**
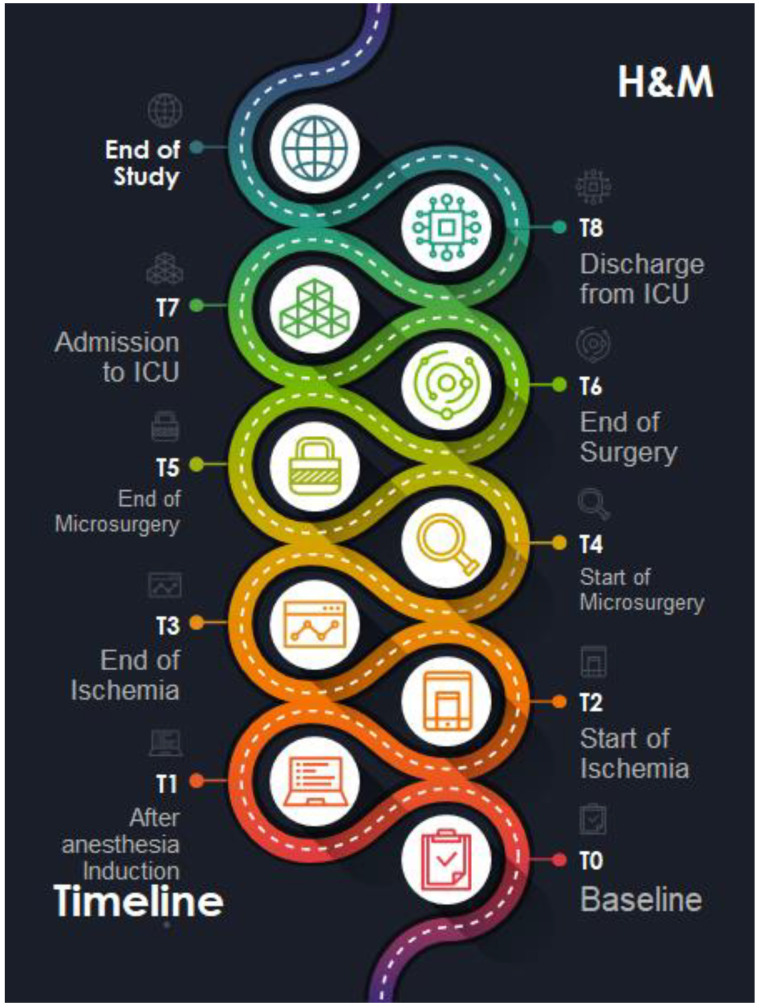
The timeline starts at baseline (T0), then moves through various stages of the surgical process, including anesthesia induction, ischemia, microsurgery, and ends with the patient’s discharge from the surgical ICU.

Continuous variables will be presented as mean (±standard deviation) or median (interquartile range: 25th–75th percentile), while categorical variables will be presented as numbers and percentages. Differences in hemodynamic parameters (macro and microcirculation) will be assessed at the following time points: baseline (T0), after anesthesia induction (T1), at the start of ischemia (T2), at the end of ischemia (T3), at the start of microsurgery (T4), at the end of microsurgery (T5), at the end of surgery (T6), at admission to ICU/SUB (T7), and on discharge from surgical ICU (T8) (Figure 1). Statistical analysis will be performed using the SAS software. Pearson’s correlation coefficient will be used to assess the association between variations in macro- and microcirculatory parameters at different time points (T1, T2, T3, T4, T5, T6, T7, T8) and their baseline values (T0). A simple logistic regression model will be used to evaluate the association between variations in macro- and microcirculatory parameters and the development of postoperative complications. Adjustments for multiple testing will also be applied to the *p*-values using Bonferroni corrections. Statistical significance will be set at a two-tailed *p*-value < 0.05.

## 3. Expected Results

The study aims to evaluate the coupling between macrocirculation and microcirculation in major demolitive–reconstructive surgeries of the head and neck region. By using pulse contour monitors and the IKORUS system, the study will assess hemodynamic parameters and microcirculatory changes during different phases of surgery. The results are expected to provide insights into the impact of these surgical procedures on both macro- and microcirculation. Additionally, the study will investigate the association between variations in hemodynamic parameters and the development of postoperative complications. These findings can contribute to improving patient care and optimizing surgical outcomes in this patient population.

## Data Availability

No new data were created or analyzed in this study. Data sharing is not applicable to this article.
